# Atlanta Medical College Clinic—Service of J. G. Westmoreland, M.D.

**Published:** 1877-11

**Authors:** 


### ATLANTA MEDICAL COLLEGE CLINIC—SERVICE OF PROF. J. G. WESTMORELAND.

REPORTED BY S. W. JOHNSON.

October 9, 1877.—Charles Glasgow, colored, æt. 37 years, formerly porter for a wholesale dry goods house, but now unable to do any work.

Patient states that the first of last year he had a sore on the penis, which continued for about one month. The usual secondary symptoms of syphilis, which generally appear about the second or third month, such as sore throat and eruptions of the skin, are not remembered by the patient. He now exhibits an ulcer, with enlargement of the upper part of the sternum, and another over the lower part of the lumbar spine. These, as you observe, are of the indolent chronic variety, evidently the result of syphilitic contamination.

The irregular treatment he received was not sufficient to rid the system of the virus, and now he is the subject of tertiary symptoms. These are often deferred for a much longer time after the disease is contracted. Very often five and even ten years are required to develop the condition we here find produced in less than two; and from the history given, it evidently appeared after the lapse of less than one year. It appears from his statement, and from the clinical records, that last winter he was treated in the surgical clinic of this college for tertiary syphilis. Although the patient has no recollection of any difficulty which will answer for secondary symptoms, it is by no means certain that he did not have them. A sore throat and eruptions of mild character may have existed without impressing the patient with their importance sufficiently to remember them.

The treatment instituted last winter in the surgical clinic, though, as appears from the history, beneficial in moderating the symptoms, was not continued sufficiently long to give decided relief.

This period of the disease is subject to the same constitutional or elective treatment as the former stages, except that the quantity of iodine must be much larger in each dose; whereas, ten grains make the usual dose in the secondary stage, forty and even sixty to eighty are required in the tertiary. Mercury, which had, until recently, gone out of use in this stage, is now being used exclusively by some, and in very small quantity. An amount little above the homœopathic dose is given regularly for a year or two, with the effect, it is said, of giving immunity against the reappearance of tertiary symptoms. While we are not inclined to rely on this remedy to give prompt relief from the pain and ulceration we frequently find in this stage of syphilis, we must admit that recent investigations have proved that mercury has action not before known; and as it has, from time immemorial, been used with the prospect of success in the primary stage, as we suppose with the effect of eliminating the virus, why may it not do the same at any stage? This remedy has been withheld in the tertiary stage, under the belief that it impoverishes the blood, and, therefore, should not be used when the system requires building up, as is supposed to be necessary under such circumstances. It has been demonstrated, however, by recent experiments, that while fibrine in the blood may be lessened, the red corpuscles are always increased by small doses of mercury. If this be true—and we do not feel warranted in denying it—there is not the danger from impoverishment that the profession suspected.

Another question, gentlemen, to which I invite your attention, and which I propose to settle as far as possible in the treatment of this and other cases, is, whether other preparations of iodine are equally efficient in the relief of tertiary symptoms, as that of the iodide. My opinion is, that iodine is the catalytic or destroyer of syphilitic virus, and that in all probability the tincture will answer the purpose. With the view of testing the correctness of this theory, I propose to use the tincture exclusively in this case. We will suppose a drop of the tincture about the equivalent of one grain of the iodide, and prescribe it accordingly. There has been objections to the administration of large doses of the tincture, on account of its supposed poisonous effects, manifested in a burning sensation of the stomach, stricture of the fauces, etc., but I think we shall be able to satisfy ourselves that when properly diluted no such difficulty will exist. The patient will probably attend the clinic regularly for some time, so that the effect of this preparation of iodine may be witnessed. I make the following prescription:

℞.—Tinct. Iodine.

Whisky, . . . . . . . aa. f ℥j.

Mix.

S.—One teaspoonful three times a day, in a half pint of water.

In this dose, about thirty drops of the tincture will be taken, and the half pint of water will sufficiently dilute it to prevent any seriously injurious or unpleasant effects upon the stomach or throat.

The mixture with whisky is ordered in order to make the dose amount to a teaspoonful, thereby avoiding the trouble and uncertainty of dropping every time the dose is taken, and to preserve the solution, which would be interfered with by mixing the tincture with water instead of whisky.

Oct. 15. The patient reports unpleasant sensation in the stomach and throat soon after taking the dose prescribed on the 9th inst., but has continued its use, notwithstanding. Now, in order to obviate this difficulty, I require him to take an additional amount of water after swallowing the half pint mixture. The amount of iodine will also be increased, as in the following:

℞.—Tinct. iodine, . . . . . . f ℥jss.
Whisky, . . . . . . . f ℥i.
Mix.

S.—One teaspoonful three times a day, in half pint of water, followed by an additional drink of water.

Oct. 22. The patient reports freedom from the unpleasant effects of the iodine upon the stomach and throat, since an additional amount of water was directed, notwithstanding the increased quantity of the remedy in the dose.

I shall now still further increase the quantity of iodine, and instead of whisky to make up the teaspoonful, shall use the tincture of gentian, which will equally well preserve the perfect solution, and at the same time invigorate the stomach. The following prescription affords about forty-six drops tinct. iodine at the dose:

℞.—Tinct. iodine, . . . . . . f ℥iij ss.
Tinct. gentian, . . . . . f ℥i.
Mix.

S.—One teaspoonful three times a day, in half pint of water, followed by a few additional swallows of water.

